# Effect of Experimental Pain Induced by Electrical Stimulation on Static and Dynamic Balance Test Results

**DOI:** 10.7759/cureus.80537

**Published:** 2025-03-13

**Authors:** Ryosuke Tozawa, Tsubasa Kawasaki

**Affiliations:** 1 Department of Physical Therapy, Faculty of Health Science, SBC Tokyo Medical University, Urayasu, JPN; 2 Department of Physical Therapy, School of Health Sciences, Tokyo International University, Kawagoe, JPN

**Keywords:** dynamic balance test, electrical stimulation, experimental pain, standing sway, static balance test

## Abstract

Background: Patients often report experiencing pain, which can impose cognitive demands and affect balance. This cognitive burden may impact the ability to maintain a stable standing position during dual-tasking. This study aimed to investigate standing postural sway in healthy adults under electrical stimulation-induced pain.

Methods: Twenty-one participants, including 12 men and 9 women, were included in this study. Static and dynamic balance tests were conducted using a force plate. Electrodes from the Silver Spike Point electrotherapy device were attached to the inferior end of the ulnar styloid. The static balance test involved standing on the force plate, while the dynamic balance was assessed through a cross-test under two conditions: with and without electrical stimulation. Electrical stimulation was delivered using a transcutaneous electrical nerve stimulation unit at an intensity that elicited a pain level of 5 on a numerical rating scale, as reported by the participants. Four tests, including static balance (with stimulus), static balance (without stimulus), cross (with stimulus), and cross (without stimulus), were each performed twice in a random order. Statistical analyses were performed to compare all variables between the control (without stimulus) and intervention (with stimulus) groups.

Results: The static balance test showed a significantly reduced sway path length (37.16 cm without stimulation vs. 33.29 cm with stimulation). The dynamic balance test (cross-test) revealed no significant differences.

Conclusions: Healthy adult participants under electrical stimulation-induced pain had lower static standing sway. However, the dynamic balance test did not show significant changes even with the addition of electrical stimulation.

## Introduction

Many daily tasks, such as dressing, washing clothes, and cleaning, are performed standing. These tasks require the use of cognitive function while maintaining a standing posture. The simultaneous execution of multiple tasks is referred to as multitasking. When concurrent tasks are executed, the performance of one or both tasks often declines compared to single-task execution [1-3]. Coordinating concurrent motor and cognitive tasks at once interference, including maintaining a standing position, is a significant clinical concern due to an increased risk of falls [4].

In clinical practice, many patients complain of pain, which is cognitively demanding because it spontaneously draws attention to the location and intensity of the pain [5,6]. Therefore, when experiencing pain, maintaining a standing position while coordinating concurrent motor and cognitive tasks is more challenging. Previous studies have examined the effects of pain on standing postural balance in patients with various conditions, such as chronic low back pain [7], patellar tendonitis [8], knee osteoarthritis [9-11], hip osteoarthritis [9,12], and rheumatoid arthritis [13]. However, these conditions involve not only pain but also factors such as changes in alignment, muscle weakness, and limited joint range of motion, all of which can directly affect standing postural balance. Therefore, determining the impact of pain alone on standing postural balance is challenging. Suda et al. investigated the double-task interference of pain sensation and reported the effects of intramuscular hypertonic saline injection into the medial and lateral vastus muscles on standing postural balance during the onset of acute pain [14]. However, the vastus medialis is reportedly to have cooperative structures with the gastrocnemius and extensor digitorum longus muscles during upright postural control, resulting in muscle synergy [15,16]. Direct intramuscular injections into the vastus medialis may impair both its normal function and the muscle synergy required for upright balance. Consequently, such studies may not clearly reveal the direct effects of pain onset on postural control. While previous studies have investigated the effect of pain on balance, the immediate effects of pain onset in the context of intact muscle function have not been specifically examined.

Therefore, this study employed a method of inducing localized pain perception through electrical stimulation at a site unaffected by standing posture to investigate the impact of the perceived cognitive load of pain on posture. Currently, whether pain perception has a positive or negative effect on standing postural sway remains unknown. Pain perception can interfere with the performance of concurrent tasks, which may lead to increased postural sway or enhanced automaticity of postural maintenance by shifting attention to pain. This study aimed to investigate the effects of electrical stimulation-induced pain on standing postural sway in healthy adults. Although it is ultimately essential to explore this within a multitasking context, the present study initially focused on a dual-task paradigm.

## Materials and methods

Study design and setting

This cross-sectional study used an electrical stimulation method of inducing pain at a site unaffected by standing posture to investigate the impact of the perceived cognitive pain load on posture. The study was approved by the Ethics Review Committee of SBC Tokyo Medical University (approval number 20-08, approval date July 8, 2020). This work was conducted at SBC Tokyo Medical University (formerly Ryotokuji University) in Urayasu City, Chiba, Japan, on November 9-24, 2021.

Participants

This study involved 21 individuals (12 men and 9 women) with no history of orthopedic disease or dysfunction. The means ± standard deviation (SD) for age, height, and weight were 21.4 ± 0.8 years, 165.8 ± 9.0 cm, and 59.7 ± 9.3 kg, respectively. All participants provided written informed consent prior to enrollment according to the Declaration of Helsinki.

Procedures

**Figure 1:**
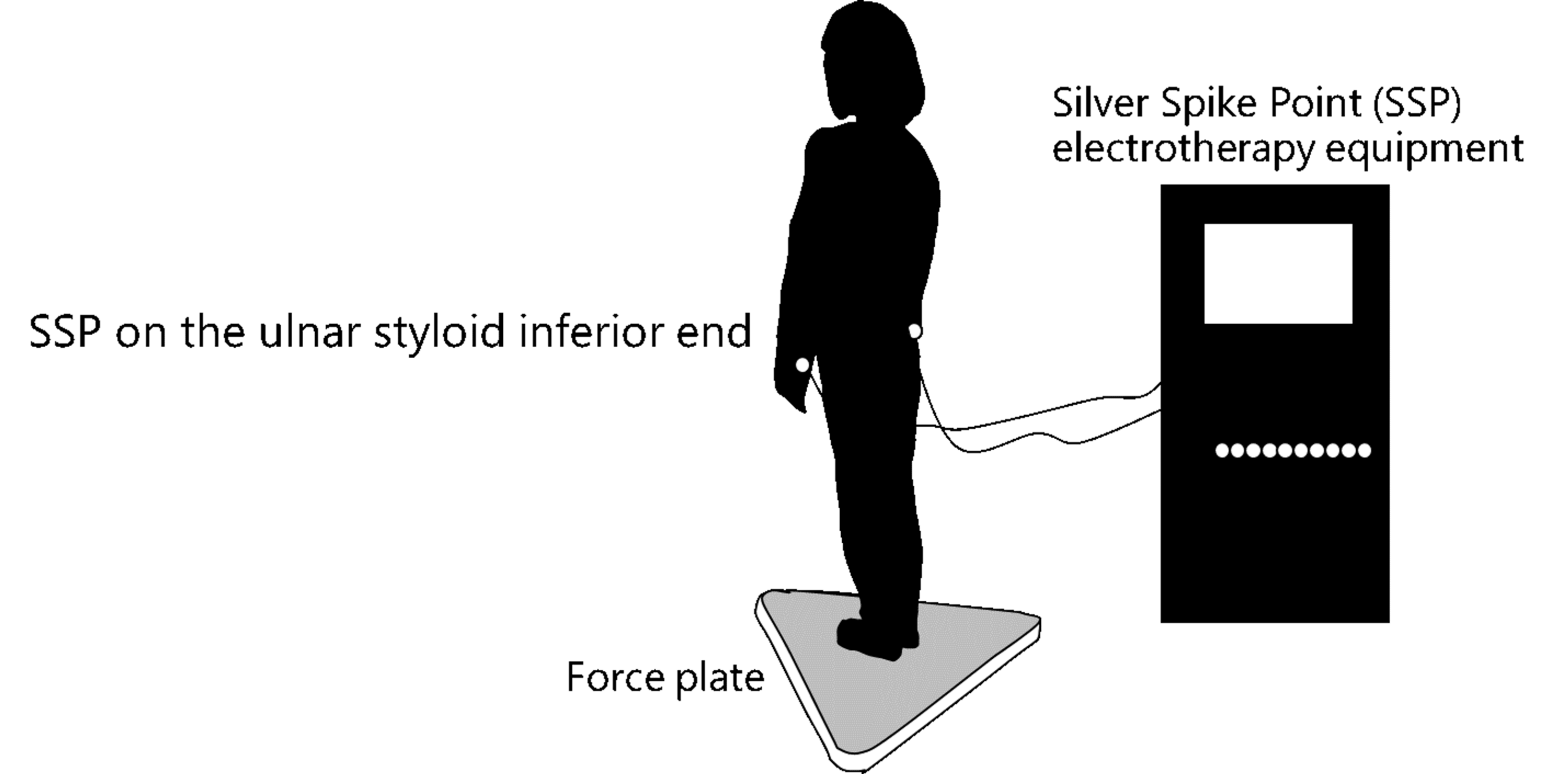
Measuring equipment Credit: Figure prepared by Ryosuke Tozawa using Microsoft PowerPoint 2016

Static and dynamic balance tests were performed using a force plate (BALANCECORDER BW-6000; Anima Inc., Japan). In addition, for each balance test, electrodes from the Silver Spike Point (SSP) electrotherapy equipment (ASTEO TS-2000, Nihon Medix Co. Ltd., Japan) were attached to the inferior end of the ulnar styloid (Figure 1).

**Figure 2:**
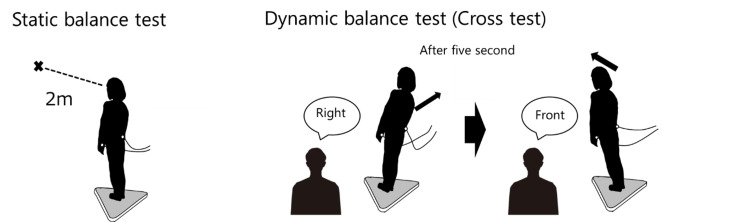
Measurements of each balance test Credit: Figure prepared by Ryosuke Tozawa using Microsoft PowerPoint 2016

The static balance test was performed in a standing position on the force plate, with participants instructed to stand for 40 seconds while looking at an “X” mark 2 meters ahead (Figure 2). The dynamic balance test was performed using a cross-test, during which participants shifted their weight to the front, back, left, and right on the force plate (Figure 2). The direction of the weight shift was randomly instructed by the examiner every 5 seconds, for a total of eight shifts (two in each direction). Participants were instructed to shift their weight as much as possible in the given direction. Both static and dynamic balance tests were performed under two conditions: with and without electrical stimulation. Stimulation during the experiment was delivered using the SSP device, with the stimulation intensity adjusted to elicit a pain level of 5 on a numerical rating scale (NRS), as reported by the participant. During the pre-study period, the inferior end of the ulnar styloid was adapted as the site of minimal muscle contraction. Four tests, including static balance (with and without stimulation) and cross (with and without stimulation), were each performed twice in a random order. The measurement variables for each balance test included sway path length (SPL), anterior-posterior length (APL), medial-lateral length (MLL), and total area of excursion (TAE). To assess workload, the National Aeronautics and Space Administration Task Load Index (NASA-TLX) questionnaire was used [17]. For the evaluation, raw NASA-TLX data, which has an average workload score ranging from 1 to 100, was used. Average workload scores were calculated by multiplying each rating by 5 [18]. The NASA-TLX was translated into Japanese for this study. It comprises six dimensions for assessing mental workload: mental demand (MD), physical demand (PD), temporal demand (TD), personal performance (OP), effort (EF), and frustration (FR).

Statistical analyses

Data obtained from measurements were noted on a Microsoft Excel 2016 (Microsoft Corp., Redmond, USA) spreadsheet. Data are expressed as means ± SD or medians (25th-75th percentile). The average of the two measurements was used as the representative value. Statistical comparisons of the measurement results between the control (without stimulus) and intervention (with stimulus) groups were performed using the t-test and Wilcoxon test. A two-sided p < 0.05 was considered statistically significant. All analyses were conducted using Modified R Commander 2.8.1 (CRAN, Freeware) for Windows [19].

## Results

All participants in the experiment perceived the electrical stimulation as pain. No significant differences in the amount of electrical stimulation were observed between the first and second sessions during the resting, standing, or cross-tests (Table 1).

**Table 1:** Stimulus values Unit: mV, mean (SD), p-values determined via t-test. SD: standard deviation

Test	First time		Second time		Statistic (t value)	Effect size (r-value)	p-value
Static balance test	57.71	(24.89)	56.19	(25.56)	-0.20	0.04	0.85
Dynamic balance test	58.38	(25.86)	57.29	(24.16)	0.83	0.18	0.45

In the static balance test, the SPL was significantly lower with stimulation (33.29 cm) than without stimulation (37.16 cm). The dynamic balance test (cross-test) revealed no significant differences under any condition (Table 2).

**Table 2:** Balance status *median (IQR), p-values determined via Wilcoxon test. ^†^ Mean (SD), p-values determined via t-test. SPL: sway path length, APL: anterior-posterior length, MLL: medial-lateral length, TAE: total area of excursion; IQR: interquartile range; SD: standard deviation

Test	Variables	Without stimulus (n = 21)		With stimulus (n = 21)		Statistic (t value or W value)	Effect size (r-value)	p-value
Static balance test	SPL^*^, cm	37.16	(32.29-40.22)	33.29	(30.09-35.58)	W = 48	0.51	0.02
	APL^*^, cm	24.22	(19.97-24.09)	21.85	(20.24-26.46)	W = 81	0.26	0.24
	MLL^*^, cm	20.55	(19.71-24.06)	19.41	(18.27-21.92)	W = 49	0.50	0.02
	TAE^*^, cm^2^	1.58	(1.19-2.48)	1.31	(0.97-1.61)	W = 38	0.59	0.01
Dynamic balance test	SPL^*^, cm	177.79	(168.07-196.03)	177.38	(163.94-188.93)	W = 96	0.15	0.52
(Cross-test)	APL^*^, cm	116.01	(107.05-125.34)	114.00	(105.93-124.15)	W = 125	0.07	0.76
	MLL^*^, cm	112.17	(106.88-124.46)	110.33	(102.77-117.55)	W = 80	0.27	0.23
	TAE^†^, cm^2^	78.10	(21.05)	71.09	(20.42)	t = 0.23	0.05	0.82

In the static balance test, the mean percentage of attention directed toward the forward gaze point was significantly higher without stimulation (46.29%) than with stimulation (36.76%). The median percentage of attention directed toward the electrode attachment point was 0.00% without stimulation but was significantly higher with stimulation (10.00%). Similarly, in the dynamic balance test with stimulation, the dispersion of attention toward the electrode-applied sites significantly increased, while attention directed to other sites significantly decreased (Table 3).

**Table 3:** Distribution of attention Unit: percent *Median (IQR), p-values determined via the Wilcoxon test. ^†^Mean (SD), p-values determined via t-test. IQR: interquartile range; SD: standard deviation

Test	Variables	Without stimulus (n=21)		With stimulus (n=21)		Statistic (t value or W value)	Effect size (r-value)	p-value
Static balance test	Point of gaze^†^	46.29	(27.83)	36.76	(19.06)	t = -2.79	0.53	0.01
	Plantar surface^†^	40.95	(27.06)	40.38	(21.90)	t = -0.20	0.04	0.85
	Point of electrodes^*^	0.00	(0.00-10.00)	10.00	(10.00-20.00)	W = 141	0.67	< 0.01
	Others^*^	0.00	(0.00-0.00)	0.00	(0.00-0.00)	W = 0	0.29	0.50
Dynamic balance test	Plantar surface^†^	50.19	(26.94)	51.05	(21.20)	t = 0.24	0.05	0.82
(Cross-test)	Point of electrodes^*^	0.00	(0.00-5.00)	10.00	(10.00-20.00)	W = 171	0.79	< 0.01
	Others^*^	47.50	(26.25-51.33)	31.50	(20.00-37.62)	W = 6.5	0.71	< 0.01

**Figure 3:**
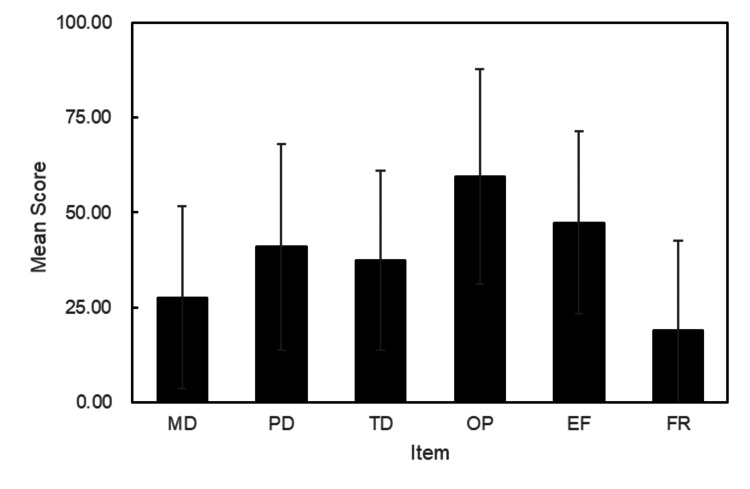
Score of NASA-TLX MD: mental demand; PD: physical demand; TD: temporal demand; OP: personal performance; EF: efficiency; FR: frustration; NASA-TLX: National Aeronautics and Space Administration Task Load Index Credit: Figure prepared by Ryosuke Tozawa using Microsoft PowerPoint 2016

The mean (SD) scores for each item in the raw NASA-TLX dataset were 27.62 (23.99) for MD, 40.95 (27.11) for PD, 37.38 (23.58) for TD, 59.52 (28.28) for OP, 47.38 (24.08) for EF, and 19.05 (23.58) for FR, as shown in Figure 3.

## Discussion

To investigate the effects of cognitive pain on stability, pain was induced using electrical stimulation applied to a site unaffected by posture. The results showed a reduction in static standing sway in healthy adults when pain was induced by electrical stimulation. However, the dynamic balance test did not show significant changes with the addition of electrical stimulation.

In automated standing positions, such as static standing, there is a possibility that the amount of sway may be reduced. However, in non-automated standing positions, the amount of sway may not be affected by the pain stimulus. Previous studies have shown that double-task interference increases sway [20-22]. However, our results do not support these findings. Although the reason is unclear, two factors may facilitate the automation of standing posture.

The first factor is the strength of the cognitive load induced by pain from electrical stimulation. However, in our study, no statistically significant differences were observed in the electrical stimulation between the static and dynamic balance tests, suggesting that the cognitive load was consistent across conditions. In this study, the degree of cognitive effort was evaluated using the NASA-TLX [17,18]. Rendell et al. reported that learners assigned to random practice had higher levels of MD, EF, and FR than those in block practice, suggesting that differences in cognitive load may cause differences in NASA-TLX subscales [23]. Although few studies have applied the NASA-TLX to standing tasks, a study using the Biodex balance system without cognitive load reported MD, PD, TD, OP, EF, and FR values of 47.3, 70.3, 44.0, 33.3, 68.0, and 29.7, respectively, for the most stable standing task [24]. In contrast, the MD, EF, and FR values reported in our study were all lower than those in the previous study, presumably due to less cognitive load from the experimental pain, which may explain the lack of dual-task interference.

The second factor may be attentional dispersion. Questionnaire results showed significantly increased attention to the electrode sites in the stimulation group, with decreased attention to the gaze and other points. Woollacott and Shumway-Cook reviewed attention during postural control and reported that attentional demands vary based on the difficulty and type of the task [25]. Improved postural stability during simple cognitive tasks may reflect a shift toward subcortical structures, thereby permitting more automatic control of upright posture [25,26]. The small cognitive task involving painful stimuli in this study may have moderately distributed attention, thereby facilitating the automation of upright postural control. Additionally, attention to body sway has been shown to inhibit automation and reduce the efficiency of maintaining a standing posture [27]. Therefore, the task of standing straight up and looking at the gaze point may have promoted active postural maintenance. On the other hand, the cross-test for dynamic balance was an intentionally controlled exercise that was not automated. Therefore, the stimulus was considered to have no influence on automaticity. Although pain is often regarded as negative and needs to be eliminated [28-30], our results suggest that pain may be positive for automated postural control and should be considered with respect to changes in body sway in pain management.

This study has several limitations, including the use of electrical stimulation to induce experimental pain. Our results may not directly correspond to pain in a clinical setting, as the nature and course of pain differ. In addition, factors contributing to the reduction in center of gravity sway in the static standing position were not possible to identify. Future research should be conducted on patients experiencing pain in clinical practice. In addition, factors contributing to the reduction in center of gravity sway in the static standing position should be clarified. Furthermore, this study employed the cross-test as a dynamic balance assessment; however, its relatively low level of difficulty may have influenced the results. More challenging balance tests could yield different outcomes, necessitating further investigation. Moreover, while this study focused on a dual-task paradigm, daily life often involves multitasking situations. Therefore, future studies should explore these more complex contexts to better reflect real-world conditions.

## Conclusions

The investigation into the impact of the perceived cognitive load of pain on posture, using pain induced by electrical stimulation, showed a reduction in static standing sway in healthy adults. In addition, the dynamic balance test did not show significant changes. This result may reflect an enhancement in the automaticity of postural maintenance by diverting attention to pain. The facilitation of standing posture automation may be attributed to less cognitive load associated with the experimental pain, which could explain the lack of dual-task interference. Therefore, the small cognitive task involving painful stimuli in this study may have moderately distributed attention and facilitated the automation of upright postural control. Future research should be conducted on patients experiencing pain in clinical practice to confirm these findings. In addition, factors contributing to the reduction in center of gravity sway in the static standing position should be clarified.
